# The acoustic repertoire of the Atlantic Forest Rocket Frog and its consequences for taxonomy and conservation (*Allobates*, Aromobatidae)

**DOI:** 10.3897/zookeys.692.12187

**Published:** 2017-08-21

**Authors:** Lucas Rodriguez Forti, Thaís Renata Ávila da Silva, Luís Felipe Toledo

**Affiliations:** 1 Laboratório Multiusuário de Bioacústica (LMBio) e Laboratório de História Natural de Anfíbios Brasileiros (LaHNAB), Departamento de Biologia Animal, Instituto de Biologia, Universidade Estadual de Campinas, Caixa Postal 6109, 13083-970, Campinas, São Paulo, Brazil

**Keywords:** Anuran communication, bioacoustics, conservation, female vocalization, taxonomy

## Abstract

The use of acoustic signals is a common characteristic of most anuran species to mediate intraspecific communication. Besides many social purposes, one of the main functions of these signals is species recognition. For this reason, this phenotypic trait is normally applied to taxonomy or to construct evolutionary relationship hypotheses. Here the acoustic repertoire of five populations of the genus *Allobates* from the Brazilian Atlantic Forest are presented for the first time, on a vulnerable to extinction Neotropical taxon. The description of males’ advertisement and aggressive calls and a female call emitted in a courtship context are presented. In addition, the advertisement calls of individuals from distinct geographical regions were compared. Differences in frequency range and note duration may imply in taxonomic rearrangements of these populations, once considered distinct species, and more recently, proposed as a single species, *Allobates olfersioides*. Calls of the male from the state of Rio de Janeiro do not overlap spectrally with calls of males from northern populations, while the shorter notes emitted by males from Alagoas also distinguishes this population from the remaining southern populations. Therefore, it is likely that at least two of the junior synonyms should be revalidated. Similarities among male advertisement and female calls are generally reported in other anuran species; these calls may have evolved from a preexisting vocalization common to both sexes. Male aggressive calls were different from both the male advertisement and female calls, since it was composed by a longer and multi-pulsed note. Aggressive and advertisement calls generally have similar dominant frequencies, but they have temporal distinctions. Such patterns were corroborated with the Atlantic Forest Rocket Frogs. These findings may support future research addressing the taxonomy of the group, behavioral evolution, and amphibian conservation.

## Introduction

Acoustic communication is the most used channel of intraspecific information transference in anurans (Walkowiak 2007; Wells 2007). Diverse social functions are mediated by such acoustic signals, for example during territorial conflicts and mate attraction (Wells 2007; Toledo et al. 2015). Sounds are generally emitted by males, which present complex vocal apparatus, generally including the vocal sac, an adaptive structure for resonance (Duellman and Trueb 1986; Wells and Schwartz 2007), and multimodal communication (Starnberger et al. 2014). The mating system in anurans is the most likely explanation for this fact, since females usually represent the selective sex and have to discriminate among potential mates using acoustic evidence from male calls (Wells 1977). However, even without a vocal sac, some females of different species are able to produce sounds, mainly during short-range interactions (Preininger et al. 2016). Females basically use acoustic signals to advertise receptivity for a candidate male during courtship (Márquez and Verrelli 1991; Bosch 2002), but sometimes, female calls are used in aggressive (Preininger et al. 2016) or defensive contexts (Toledo and Haddad 2009). Considering the intraspecific social functions, these acoustic signals potentially carry important evidence of species recognition, and the appropriate calls description is useful for taxonomic decisions and future evolutionary studies (Robillard et al. 2006; Padial et al. 2008; Köhler et al. 2017).

Information that elucidates taxonomy is especially useful in the case of the Atlantic Forest populations of *Allobates* Zimmermann & Zimmermann, 1988. This genus has been facing some taxonomic instability: based on morphology, a previous study (Verdade and Rodrigues 2007, Frost 2016) placed three other available specific names for these geographically widespread populations into junior synonymy of *A. olfersioides* (Lutz, 1925): *A. alagoanus* (Bokermann, 1967), *A. capixaba* (Bokermann, 1967), and *A. carioca* (Bokermann, 1967). However, other authors still considered these three disconnected populations as different species, *A. olfersioides* in the state of Rio de Janeiro, *A. capixaba* in the states of Espírito Santo, and *A. alagoanus* in the state of Alagoas (e.g., Haddad et al. 2013, MMA 2014). The clarification of whether these populations are distinct species or lineages of the same species is essential as, if they are considered apart, the different populations are threatened (*A. olfersioides* is VU) or data deficient (*A. alagoanus* and *A. capixaba*) (MMA 2014). If all populations are considered as one species, the conservation status of the species may change.

These species are morphologically cryptic, the argument that justified the synonymy (Verdade and Rodrigues 2007). Nevertheless, acoustic traits have often revealed differences among morphologically cryptic species (e.g. Toledo et al. 2007, Köhler et al. 2015, Andrade et al. 2016). Especially for these populations, some acoustic differences were suggested (see Bokermann 1967), but never tested. Therefore, a comparative analysis of the acoustic communication among different populations would enlighten the group taxonomy. In spite of this, populations of the Atlantic Forest *Allobates* are showing evidence of decline in the states of Rio de Janeiro and Espírito Santo (Weygoldt 1989, Verdade 2010, Carvalho et al. 2017), and hence, multiple acoustic recordings are scarce.

Therefore, in trying to provide further information on the acoustic signals of these populations, we collected recordings of the two distinct regions: Southeast and Northeast Brazil. These calls, emitted in three different social contexts, are described, and comparisons are made between the advertisement calls of individuals from five populations. This comparison exposed striking differences between some populations, indicating three putative species.

## Materials and methods

**Figure 1.**
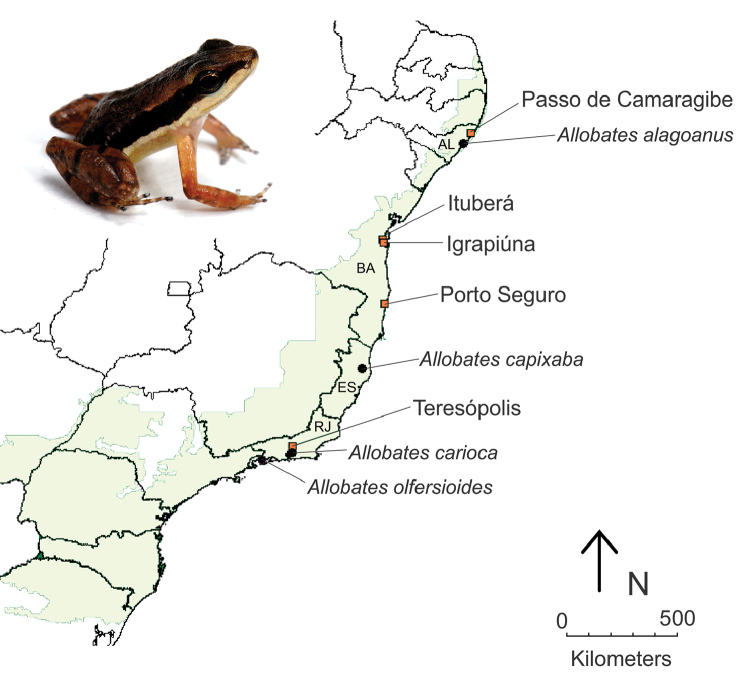
Sampled *Allobates* populations (orange squares - indicating the municipality name) and type localities of four available specific names for the Atlantic forest populations of the genus *Allobates* (black circles - indicating their specific names). Brazilian state names are abbreviated: AL - Alagoas, BA - Bahia, ES - Espírito Santo, and RJ - Rio de Janeiro. The light green shade indicates the original distribution of the Atlantic Forest. The upper left photograph of an adult male *Allobates* was taken in Igrapiúna, BA.

Sixteen audio files were obtained with calls emitted in three different social contexts: (1) advertisement calls; (2) territorial calls; and (3) female amplectant calls (*sensu* Toledo et al. 2015). These recordings come from populations of five municipalities from southeastern and northeastern Brazil (Figure 1).

Our sample from southeastern Brazil was based on the advertisement calls of one male from Teresópolis, Rio de Janeiro, recorded on 14 Dec 1977 by Ronald W. Heyer (microphone and recorder data are not available) and deposited in Fonoteca Neotropical Jacques Vielliard, FNJV (FNJV32824). Recordings from Bahia come from 12 males from the municipalities of Igrapiuna (n = 9 males), Ituberá (n = 1 male), and Porto Seguro (n = 1 female and 2 males). Using an Edirol recorder and a Sony microphone, recordings from Igrapiuna were obtained in August to September 2008, and May to July 2009, while the male from Ituberá was recorded on 04 April 2005. These audio files, with advertisement and territorial calls, can be accessed upon request in the sound collection of the Universidade Estadual de Feira de Santana, following the codes: 100.18, 100.203, 100.206, 100.209, 100.218, 100_324, 100_325, 100_326, 100_334, 100_335, 100_287. We deposited the same files referring to their original collection (mentioned above) in FNJV (FNJV 33311-33320). The calls from Porto Seguro were recorded with a DAT recorder on February 2002 at Reserva Particular Estação Veracel. Female calls were recorded while in amplexus with a conspecific male. The observer was 1 m from the calling female. This recording is also deposited in FNJV (FNJV32825). Advertisement calls of the two males from Bahia are deposited in the Scientific Collection of Amphibians Vocalizations from the National Museum of Rio de Janeiro with the following access codes: MNVOC_57_04 and MNVOC_57_06. Two males were recorded between 12 and 14 September 2004, at municipality of Passo de Camaragibe, Alagoas, Brazil. These individuals were on the forest floor, approximately 50 m from a rivulet, inside a forest remnant, nearly 100 m from the coastal shore. Advertisement and territorial calls of two males were recorded using a Marantz cassette tape recorder (PMD222) equipped with an external directional microphone (Audiotecnica AT835b) positioned approximately 50 cm from the calling male. Recordings were deposited in FNJV (FNJV12681 and 12685).

All recordings were digitized at 22 kHz of frequency sampling and 16 bits of resolution for standardize the analyzed audio files. Vocalizations were analyzed with Raven Pro 1.4 (Bioacoustic Research Program, 2011). We analyzed such acoustic properties: Note duration (s), Minimum frequency (Hz), Peak of dominant frequency (Hz), Maximum frequency (Hz), and Frequency bandwidth (as maximum subtracted by minimum) (Hz). Spectral measurements were obtained using a FFT (Fast Fourier Transform) of 1024. For acoustic measurements, we selected the calls using the spectrogram. The acoustic traits analyzed and the respective Raven functions used for measurements are present in the Suppl. material 1, Table S1. Figures were prepared with FFT of 256, with 50 % of overlap in a Hann window. Power spectrums for individual notes were generated in the software Goldwave v6.24, using the spectrum filter function. No filtering was applied in the spectrograms.

One-Way Analysis of Variance (ANOVA) was performed with post hoc test of Fisher LSD (Least Significant Difference), for comparing notes duration among different populations. We carried the analysis in the software Statistica, where we adopted the significance level of 0.05.

## Results

**Figure 2.**
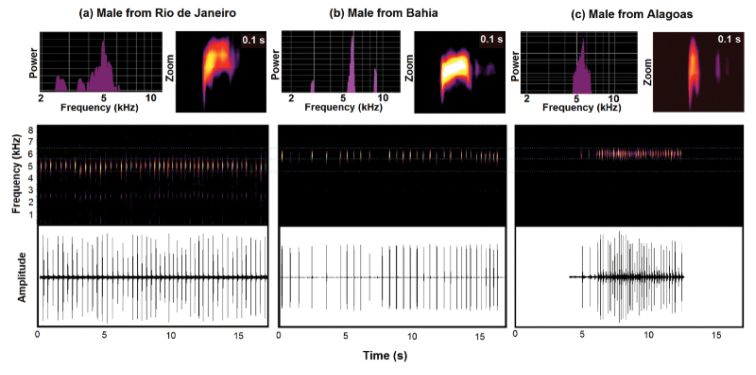
Power spectrum (above left), spectrogram (middle and detail of one note highlighted above right) and oscillogram (below) of vocalizations of three populations of the Atlantic Forest Rocket Frogs: Rio de Janeiro, state of Rio de Janeiro (a; FNJV32824); Igrapiuna, state of Bahia (b; FNJV33312); and Passo de Camaragibe, state of Alagoas (c; FNJV12685).

The advertisement call of all populations is composed by one single note, generally repeated inside a sequence with discrete intervals (Figure 2a). Notes are composed of one or two fused pulses.

**Figure 3.**
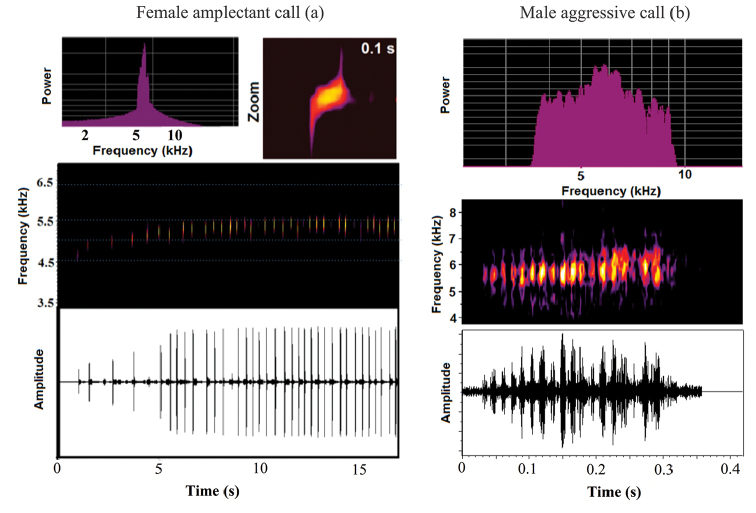
Female amplectant call of the Atlantic Forest Rocket Frog from the state of Bahia: power spectrum (above left), spectrogram (middle and detail of one note highlighted above right) and oscillogram (below) (FNJV32825) (a); and Male aggressive call of the Atlantic Forest Rocket Frog from the state of Alagoas: power spectrum (above), spectrogram (middle), and oscillogram (below) (FNJV12681) (b).

Many males (60 %) presented interval between notes decreasing along the sequence (Figure 2b and c), with stronger patterns most observed in calls of males from Alagoas. Female amplectant call sequence presents the same structure. A slight spectral modulation can be observed between notes for both call types. However, the call produced by females has more pronounced spectral modulations (Figure 3). Advertisement calls from different regions had distinct band frequency occupation: calls from Rio de Janeiro (Southeastern) are lower (not overlapping) than calls from populations of Northeastern Brazil (Figure 2). In addition, we found difference (One-Way ANOVA, F_(4,308)_ = 27.888; *P* < 0.0001, followed by Fisher LSD: Suppl. material 1, Table S2) between the note duration of the population of Alagoas and all others (shorter in Alagoas), and between the population of Ituberá and all others (intermediate between Alagoas and all others) (Suppl. material 1, Figure S1). A clinal variation on notes duration was observed, in which southern populations has longer notes than northern populations.

Male aggressive calls are different from advertisement and female amplectant calls since they are composed by a single multi-pulsed note (Figure 3). In two occasions, we registered males combining aggressive calls among sequences of advertisement calls. A detailed comparison of acoustic properties among these three types of calls and geographic variation among advertisement calls is available in the Table 1.

**Table 1.** Acoustic properties of three types of calls of the Atlantic Forest populations of *Allobates*. Values presented as mean ± SD (range) number of calls.

**Call type**	**Male advertisement call**					**Male aggressive call**		**Female amplectant call**
**Municipality (State)**	**Rio de Janeiro (RJ)**	**Igrapiuna (BA)**	**Ituberá (BA)**	**Porto Seguro (BA)**	**Passo de Camaragibe (AL)**	**Igrapiuna (BA)**	**Passo de Camaragibe (AL)**	**Porto Seguro (BA)**
**Individuals**	**1**	**9**	**1**	**2**	**2**	**1**	**1**	**1**
Interval between notes (s)	0.328 ± 0.039 (0.296 – 0.449) 15	0.666 ± 0.387 (0.203 – 2.958) 180	0.308 ± 0.035 (0.262 – 0.381) 23	0.396 ± 0.530 (0.076 – 2.921) 37	0.190 ± 0.100 (0.083 – 0.624) 48	not applicable	not applicable	0.423 ± 0.254 (0.229 – 1.163) 19
Note or call duration (s)	0.046 ± 0.003 (0.039 – 0.053) 25	0.042 ± 0.016 (0.024 – 0.089) 180	0.032 ± 0.002 (0.028 – 0.038) 23	0.044 ± 0.012 (0.030 – 0.068) 37	0.022 ± 0.006 (0.007 – 0.039) 48	0.64 ± 0.042 (0.611 – 0.706) 4	0.28 ± 0.11 (0.15 – 0.35) 3	0.032 ± 0.006 (0.026 – 0.044) 25
Minimum frequency (kHz)	4.83 ± 0.08 (4.48 – 4.91) 25	5.67 ± 0.15 (5.40 – 5.94) 180	5.82 ± 0.11 (5.55 – 6.00) 23	5.83 ± 0.08 (5.68 – 5.98) 37	5.91 ± 0.16 (5.44 – 6.31) 48	4.22 ± 0.011 (4.07 – 4.32) 4	5.41 ± 0.53 (5.36 – 5.47) 2	5.34 ± 0.76 (5.21 – 5.51) 25
Peak of dominant frequency (kHz)	4.91 ± 0.08 (4.60 – 5.06) 25	5.80 ± 0.15 (5.49 – 6.03) 180	6.01 ± 0.10 (5.64 – 6.09) 23	5.99 ± 0.04 (5.94 – 6.05) 37	6.14 ± 0.14 (5.90 – 6.35) 48	6.03 ± 0.13 (5.83 – 6.13) 4	6.04 ± 0.48 (5.55 – 6.52) 2	5.47 ± 0.78 (5.21 – 5.51) 25
Maximum frequency (kHz)	5.04 ± 0.08 (4.71 – 5.10) 25	5.86 ± 0.15 (5.55 – 6.09) 180	6.07 ± 0.06 (5.85 – 6.13) 23	6.09 ± 0.06 (5.98 – 6.30) 37	6.24 ± 0.16 (6.00 – 6.54) 48	6.56 ± 0.13 (6.37 – 6.67) 4	6.92 ± 0.42 (6.50 – 7.34) 2	5.52 ± 0.08 (5.38 – 5.72) 25
Range frequency (kHz)	0.21 ± 0.03 (0.10 – 0.28) 25	0.18 ± 0.05 (0.08 – 0.32) 180	0.24 ± 0.07 (0.08 – 0.36) 23	0.25 ± 0.06 (0.12 – 0.41) 37	0.33 ± 0.20 (0.15 – 1.07) 48	2.33 ± 0.19 (2.15 – 2.60) 4	1.50 ± 0.36 (1.14 – 1.87) 2	0.18 ± 0.05 (0.08 – 0.26) 25

## Discussion

Although the comparisons are limited due to small sample sizes, these results do not corroborate the proposed synonymy between, at least, *A. olfersioides* and the northeastern populations (Verdade and Rodrigues 2007). While the morphological data showed that these populations are cryptic, the observed variation on frequency band and note duration of the advertisement call represents a strong evidence that these populations are indeed, at least, three distinct species. This finding is in agreement with the suggested in Bokermann (1967), who indicated possible acoustics differences in regards to temporal properties (interval between notes) between *A. olfersioides* and *A. capixaba*. As we did not have access to the recordings from the state of Espírito Santo, we now face different possible taxonomic resolutions: i) the populations from Bahia are attributed to *A. capixaba*, which is distinct from the northern populations, which should be *A. alagoanus*; or ii) the populations from Bahia represents a new species, considering the population in Rio de Janeiro as *A. olfersioides*, in Alagoas as *A. alagoanus*, and in Espírito Santo as *A. capixaba* (for which we did not have call recordings). The clinal variation on note duration give margins for both interpretations.

If our evidence is questionable, due to the limited sample size, we stress the need of further recordings (mainly from Southeast populations) coupled with molecular analysis to increase resolution in the taxonomy of the Atlantic Forest Rocket Frogs. Targeting these populations in future studies are critical, as if the different populations are considered distinct species, the occurrence range of *A. olfersioides* will be restricted to the state of Rio de Janeiro, where declines and local extinctions were reported (Weygoldt 1989, Verdade 2010, Carvalho et al. 2017). In such case, *A. olfersioides* conservation status (today considered as “Least Concern”) would change to “Vulnerable”, and *A. capixaba*, and *A. alagoanus* would be considered as “Data Deficient”, as currently is classified in the Brazilian list of Threatened Species (MMA 2014). Therefore, this evaluation is not only of taxonomic interest, but may also reflect directly in anuran (and Atlantic Forest) conservation.

Some congeneric species present similar advertisement calls, with one note repeated several times in a discrete interval. It is the case in *A. algorei* Barrio-Amorós & Santos, 2009, *A. flaviventris* Melo-Sampaio, Souza & Peloso, 2013, *A. goianus* (Bokermann, 1975), *A. magnussoni* Lima, Simões & Kaefer, 2014, *A. masniger* (Morales, 2002), and *A. nidicola* (Caldwell & Lima, 2003) (Barrio-Amorós and Santos 2009, Bastos et al. 2011, Tsuji-Nishikido et al. 2012, Lima et al. 2014). However, many other species may present a complex call composed by a series of notes, as we can find in *A. brunneus* (Cope, 1887), *A. crombiei* (Morales, 2002), *A. femoralis* (Boulenger, 1884), *A. granti* (Kok, MacCulloch, Gaucher, Poelman, Bourne, Lathrop & Lenglet, 2006), *A. grillisimilis* Simões, Sturaro, Peloso & Lima, 2013, *A. kingsburyi* (Boulenger, 1918), *A. myersi* (Pyburn, 1981), *A. paleovarzensis* Lima, Caldwell, Biavati & Montanarin, 2010, and *A. talamancae* (Cope, 1875) (Kok et al. 2006, Castillo-Trenn and Coloma 2008, Simões et al. 2008, Simões and Lima 2011, Lima et al. 2012, Kaefer and Lima 2012, Simões et al. 2013, Lechelt et al. 2014, Lima et al. 2014).

A modulated interval between notes (decreasing intervals between notes during the call) observed mainly in the advertisement calls of northeast males and in the female call has not been observed in other species, except for *A. talamancae* (Lechelt et al. 2014). A similar frequency modulation observed among notes in the advertisement call of all populations was observed in the advertisement call of *A. femoralis* and *A. paleovarzensis* from Central and South-West Brazilian Amazonia (Kaefer and Lima 2012, Simões et al. 2008). Therefore, this genus is interesting and useful for testing the evolution of call structure.

Female calls (with intraspecific social context) are present in more than 50 species belonging to 12 families (Preininger et al. 2016). Our study extends this information to an additional family, providing the first female call in Aromobatidae. Females may use acoustic signals in four social contexts: advertisement, courtship, aggressiveness (Preininger et al. 2016), and defense (Toledo and Haddad 2009). Although a female calling during amplexus was registered, the signal’s social function is not clear and should still be the subject of observations and future playback experiments. Except for the pronounced spectral modulation, female calls are similar to advertisement calls of males from the same population. Emerson and Boyd (1999) suggested that in species where females vocalize during courtship, the calls might have evolved by a preexisting pathway that appears to be common to both sexes. Similarity between male and female calls are common (Bosch and Márquez 2001); however, female calls are generally less intense (in sound pressure levels) and of shorter duration than male advertisement calls (Tobias and Kelley 1987, Preininger et al. 2016).

Aggressive calls in anurans are generally different from advertisement calls in a temporal structure, and sometimes the aggressive calls are considered adjustments of the male advertisement call (Wells 2007, Wells and Schwartz 2007). As in the Atlantic Forest Rocket Frog, the aggressive calls of *A. caeruleodactylus* (Lima and Caldwell 2001) are shorter and louder than their advertisement calls (Lima et al. 2002). Therefore, it is possible that this pattern is conserved in congeneric species.

The current study presents novel acoustic information about five distinct populations of the Atlantic Forest Rocket Frogs, which may stimulate further comprehensive studies focusing on the taxonomy and systematics of the Atlantic Forest populations or even among the Neotropical congenerics. Bioacoustic characterization of different populations has been proven to be useful for both taxonomy and conservation (e.g. Méndez-Cárdenas et al. 2008, Forti et al. 2016, Köhler et al. 2017). That is especially relevant for the populations considered presently as they are already categorized as threatened (MMA 2014), population declines have been reported (Weygoldt 1989, Verdade 2010, Carvalho et al. 2017), and the Atlantic Forest itself is one of the biodiversity hotspots in the world (Myers et al. 2000).

## Supplementary materials

Supplementary material 1 The acoustic repertoire of the Atlantic Forest Rocket Frog (*Allobates*, Aromobatidae) and its consequences for taxonomy and conservation. Data type: acoustic measurements and results of specific comparing analysis Explanation note: Here, we presented complementary methodology information and additional results with statistical differences of note duration among populations. This dataset is made available under the Open Database License (http://opendatacommons.org/licenses/odbl/1.0/). The Open Database License (ODbL) is a license agreement intended to allow users to freely share, modify, and use this Dataset while maintaining this same freedom for others, provided that the original source and author(s) are credited.Lucas Rodriguez Forti, Thaís Renata Ávila da Silva, Luís Felipe Toledo

## References

[B1] Andrade FS Magalhães FM Nunes-de-Almeida CHL Veiga-Menoncello ACP Santana DJ Garda AA Loebmann D Recco-Pimentel SM Giaretta AA Toledo LF (2016) A new species of long-legged *Pseudopaludicola* from northeastern Brazil (Anura, Leptodactylidae, Leiuperinae). Salamandra 52(2): 107–124. http://www.salamandra-journal.com/index.php/home/contents/2016-vol-52/567-andrade-f-s-f-m-magalhaes-c-h-l-nunes-de-almeida-a-c-p-veiga-menoncello-et-al/file

[B2] Bastos RP Signorelli L Morais AR Costa TB Lima LP Pombal JP Jr (2011) Advertisement call of three anuran species (Amphibia) from the Cerrado, central Brazil. South American Journal of Herpetology 6: 67–72. https://doi.org/10.2994/057.006.0204

[B3] Bokermann WCA (1967) Novas espécies de *Phyllobates* do leste e sudeste brasileiro (Anura, Dendrobatidae). Revista Brasileira de Biologia 27: 349–353.

[B4] Bosch J Márquez R (2001) Female Courtship Call of the Iberian Midwife Toad (*Alytes Cisternasii*). Journal of Herpetology 35(4): 647–52. https://doi.org/10.2307/1565904

[B5] Carvalho T Becker CG Toledo LF (2017) Historical amphibian declines and extinctions in Brazil linked to chytridiomycosis. Proceedings of the Royal Society of London, B: Biological Sciences, In press. https://doi.org/10.1098/rspb.2016.2254 10.1098/rspb.2016.2254 PMC5310605 28179514

[B6] Castillo-Trenn P Coloma LA (2008) Notes on behaviour and reproduction in captive *Allobates kingsburyi* (Anura: Dendrobatidae), with comments on evolution of reproductive amplexus. International Zoo Yearbook 42: 58–70. https://doi.org/10.1111/j.1748-1090.2007.00039.x

[B7] Duellman WE Trueb L (1986) Biology of Amphibians. McGraw-Hill, NewYork, 670 pp.

[B8] Emerson SB Boyd SK (1999) Mating vocalizations of female frogs: control and evolutionary mechanisms. Brain, Behavior and Evolution 53: 187–197. 10.1159/000006594 10343085

[B9] Forti LR Costa WP Martins LB Nunes-de-Almeida CHL Toledo LF (2016) Advertisement call and genetic structure conservatism: good news for an endangered Neotropical frog. PeerJ 4: e2014: 1–16; https://doi.org/10.7717/peerj.2014 10.7717/peerj.2014 PMC4867718 27190717

[B10] Frost DR (2016) Amphibian Species of the World: an Online Reference. Version 6.0 (Date of access). Electronic Database accessible at http://research.amnh.org/herpetology/amphibia/index.html. American Museum of Natural History, New York, USA.

[B11] Haddad CFB Toledo LF Prado CPA Loebmann D Gasparini JL Sazima I (2013) Guia de anfíbios da Mata Atlântica: diversidade e biologia. Anolis books, São Paulo, 544 pp.

[B12] Kaefer IL Lima AP (2012) Sexual signals of the Amazonian frog *Allobates paleovarzensis*: geographic variation and stereotypy of acoustic traits. Behaviour 149(1): 15–33. https://doi.org/10.1163/156853912X623757

[B13] Köhler J Glaw F Pabijan M Vences M (2015) Integrative taxonomic revision of mantellid frogs of the genus *Aglyptodactylus* (Anura: Mantellidae). Zootaxa 4006(3): 401–438. https://doi.org/10.11646/zootaxa.4006.3.1 26623776 10.11646/zootaxa.4006.3.1

[B14] Köhler J Jansen M Rodríguez A Kok PJR Toledo LF Emmrich M Glaw F Haddad CFB Rödel M-O Vences M (2017) The use of bioacoustics in anuran taxonomy: theory, terminology, methods and recommendations for best practice. Zootaxa 4251(1): 1–124. https://doi.org/10.11646/zootaxa.4251.1.1 28609991 10.11646/zootaxa.4251.1.1

[B15] Kok PJR MacCulloch RD Gaucher P Poelman EH Bourne GR Lathrop A Lenglet GL (2006) A new species of *Colostethus* (Anura, Dendrobatidae) from French Guiana with a redescription of *Colostethus beebei* (Noble, 1923) from its type locality. Phyllomedusa 5: 43–66. http://www.phyllomedusa.esalq.usp.br/articles/volume5/number1/514366.pdf

[B16] Lechelt S Hödl W Ringler M (2014) The role of spectral advertisement call properties in species recognition of male *Allobates talamancae* (COPE, 1875). Herpetozoa 26: 139–150. PMC5321600 28239241

[B17] Lima AP Erdtmann LK Amézquita A (2012) Advertisement call and colour in life of *Allobates crombiei* (Morales) “2000” [2002] (Anura: Aromobatidae) from the type Locality (Cachoeira do Espelho), Xingu River, Brazil. Zootaxa 3475: 86–88. http://mapress.com/zootaxa/2012/f/z03475p088f.pdf

[B18] Lima AP Simões PI Kaefer IL (2014) A new species of Allobates (Anura: Aromobatidae) from the Tapajós River basin, Pará State, Brazil. Zootaxa 3889(3): 355–387. https://doi.org/10.11646/zootaxa.3889.3.2 25544274 10.11646/zootaxa.3889.3.2

[B19] Márquez R Verrelli P (1991) The courtship and mating of the Iberian midwife toad *Alytes cisternasii* (Amphibia: Anura: Discoglossidae). Journal of Zoology 225(1): 125–139. https://doi.org/10.1111/j.1469-7998.1991.tb03806.x

[B20] Méndez-Cárdenas M Randrianambinina B Rabesandratana A Rasoloharijaona S Zimmermann E (2008) Geographic variation in loud calls of sportive lemurs (Lepilemur ssp.) and their implications for conservation. American Journal of Primatology 9: 828–838. https://doi.org/10.1002/ajp.20554 10.1002/ajp.20554 18484626

[B21] MMA (Ministério do Meio Ambiente) (2014) Lista Nacional Oficial de Espécies da Fauna Ameaçadas de Extinção. Portaria nº 444 de 17 de dezembro de 2014.

[B22] Myers N Mittermeier RA Mittermeier CG Fonseca GAB Kent J (2000) Biodiversity hotspots for conservation priorities. Nature 403: 853–845. https://doi.org/10.1038/35002501 10706275 10.1038/35002501

[B23] Padial JM Köhler J Muñoz A De La Riva I (2008) Assessing the taxonomic status of tropical frogs through bioacoustics: geographical variation in the advertisement calls in the *Eleutherodactylus discoidalis* species group (Anura). Zoological Journal of the Linnean Society 152: 353–365. https://doi.org/10.1111/j.1096-3642.2007.00341.x

[B24] Preininger D Handschuh S Boeckle M Szatatecsny M Hödl W (2016) Comparison of female and male vocalisation and larynx morphology in the size dimorphic foot-flagging frog species *Staurois guttatus*. Herpetological Journal 26(1): 87–197. http://www.ingentaconnect.com/content/bhs/thj/2016/00000026/00000003/art00001

[B25] Pyron RA Wiens JA (2011) A large-scale phylogeny of Amphibia including over 2800 species, and a revised classification of extant frogs, salamanders, and caecilians. Molecular Phylogenetics and Evolution 61(2): 543–583. https://doi.org/10.1016/j.ympev.2011.06.012 21723399 10.1016/j.ympev.2011.06.012

[B26] Robillard T Höbel G Gerhardt HC (2006) Evolution of advertisement call signals in North American hylid frogs: vocalizations as end-products of calling behavior. Cladistics 22: 533–545. https://doi.org/10.1111/j.1096-0031.2006.00118.x 10.1111/j.1096-0031.2006.00118.x 34892895

[B27] Salthe SN Mecham JS (1974) Reproductive and courtship patterns. In: Lofts B (Ed.) Physiology of the Amphibia (vol. 2). Academic Press, New York, 309–521. https://doi.org/10.1016/B978-0-12-455402-3.50010-3

[B28] Simões PI Lima AP (2011) The complex advertisement calls of *Allobates myersi* (Pyburn, 1981) (Anura: Aromobatidae) from São Gabriel da Cachoeira, Brazil. Zootaxa 2988: 66–68. https://doi.org/10.11646/zootaxa.3889.3.2

[B29] Simões PI Lima AP Magnusson WE Hödl W Amézquita A (2008) Acoustic and morphological differentiation in the frog *Allobates femoralis*: Relationships with the upper Madeira River and other potential geological barriers. Biotropica 40: 607–614. https://doi.org/10.1111/j.1744-7429.2008.00416.xv

[B30] Simões PI Sturaro MJ Peloso PLV Lima AP (2013) A new diminutive species of *Allobates* Zimmermann and Zimmermann, 1988 (Anura, Aromobatidae) from the northwestern Rio Madeira/Rio Tapajós interfluve, Amazonas, Brazil. Zootaxa 3609(3): 251–273. https://doi.org/10.11646/zootaxa.3609.3.1 24699589 10.11646/zootaxa.3609.3.1

[B31] Starnberger I Preininger D Hödl W (2014) The anuran vocal sac: a tool for multimodal signalling. Animal Behaviour 97: 281–288. https://doi.org/10.1016/j.anbehav.2014.07.027 25389375 10.1016/j.anbehav.2014.07.027 PMC4222773

[B32] Tobias ML Kelley DB (1987) Vocalizations by a sexually dimorphic isolated larynx: peripheral constraints on behavioral expression. The Journal of Neuroscience 7: 3191–3197. http://www.ncbi.nlm.nih.gov/pmc/articles/PMC3493245/3668623 10.1523/JNEUROSCI.07-10-03191.1987 PMC3493245

[B33] Toledo LF Garcia PCA Lingnau R Haddad CFB (2007) A new species of *Sphaenorhynchus* (Anura; Hylidae) from Brazil. Zootaxa 1658: 57–68. http://www.mapress.com/zootaxa/2009/f/z02115p046f.pdf

[B34] Toledo LF Haddad CFB (2009) Defensive vocalizations of neotropical anurans. South American Journal of Herpetology 4(1): 25–42. https://doi.org/10.2994/057.004.0104

[B35] Toledo LF Martins IA Bruschi DP Passos MA Alexandre C Haddad CFB (2015) The anuran calling repertoire in the light of social context. Acta Ethologica 18(2): 87–99. https://doi.org/10.1007/s10211-014-0194-4

[B36] Tsuji-Nishikido BM Kaefer IL Freitas FC Menin M Lima AP (2012) Significant but not diagnostic: differentiation through morphology and calls in the Amazonian frogs *Allobates nidicola* and *A. masniger*. Herpetological Journal 22: 105–114.

[B37] Verdade V (2010) *Allobates olfersioides*. The IUCN Red List of Threatened Species: e.T55122A11255268. [Downloaded on 27 July 2016]

[B38] Verdade VK Rodrigues MT (2007) Taxonomic review of *Allobates* (Anura, Aromobatidae) from the Atlantic Forest, Brazil. Journal of Herpetology 41(4): 566–580. https://doi.org/10.1670/06-094.1

[B39] Walkowiak W (2007) Call production and neural basis of vocalization. In: Narins PM Feng AS Fay RR Popper AN (Eds) Hearing and Sound Communication in Amphibians. Springer, New York, 87–112.

[B40] Wells KD (1977) The social behavior of anuran amphibians. Animal Behaviour 25: 666–693. https://doi.org/10.1016/0003-3472(77)90118-X

[B41] Wells KD Schwartz JJ (2007) The Behavioral Ecology of Anuran Communication. In: Narins PM Feng AS Fay RR Popper AN (Eds) Hearing and Sound Communication in Amphibians. Springer Handbook of Auditory Research, New York, 44–86.

[B42] Weygoldt P (1989) Changes in the composition of mountain stream frog communities in the Atlantic mountains of Brazil: frogs as indicators of environmental deteriorations? Studies of Neotropical Fauna and Environment 24: 249–255. https://doi.org/10.1080/01650528909360795

