# Supplementary material for: The acoustic repertoire of the Atlantic Forest Rocket Frog and its consequences for taxonomy and conservation (Allobates, Aromobatidae)
Source: Zookeys. 2017 Aug 21;(692):141–53. doi: 10.3897/zookeys.692.12187 (PMC5672738; doi:10.3897/zookeys.692.12187)
Supplement: Supplementary material 1 — The acoustic repertoire of the Atlantic Forest Rocket Frog (Allobates, Aromobatidae) and its consequences for taxonomy and conservation. [file zookeys-692-141-s001.pdf]

Supplementary material

Table S1. List of acoustic traits analyzed and the Raven functions used for their measurements.

Acoustic trait	Raven function for measurement
Note duration (s)	Delta time
Minimum frequency (Hz)	Frequency 5% (minimum frequency, ignoring 5% below the total energy in the selected call)
Peak of dominant frequency (Hz)	Peak frequency
Maximum frequency (Hz)	Frequency 95% (maximum frequency, ignoring 5% above the total energy in the selected call)
Frequency bandwidth (as maximum subtracted by minimum) (Hz)	Bandwidth 90% (band frequency that includes 90% of the energy of the sound)

Table S2. Results of Fisher LSD analysis comparing notes duration among Atlantic Forest Rocket Frogs populations.

Interaction	Difference	Statistic test	*P* level
Igrapiúna x Ituberá	0.00997	3.452	**0.00063**
Igrapiúna x Passo de Camaragibe	0.02005	9.463	**0.E+0**
Igrapiúna x Porto Seguro	-0.00228	0.967	0.33381
Igrapiúna x Teresópolis	-0.00412	1.478	0.00251
Ituberá x Passo de Camaragibe	0.01008	3.047	0.14019
Ituberá x Porto Seguro	-0.01225	3.537	**0.00047**
Ituberá x Teresópolis	-0.01409	3.738	**0.00022**
Passo de Camaragibe x Porto Seguro	-0.02233	7.825	**8.01581E-14**
Passo de Camaragibe x Teresópolis	-0.02417	7.512	**6.24167E-13**
Porto Seguro x Teresópolis	-0.00184	0.544	0.58663

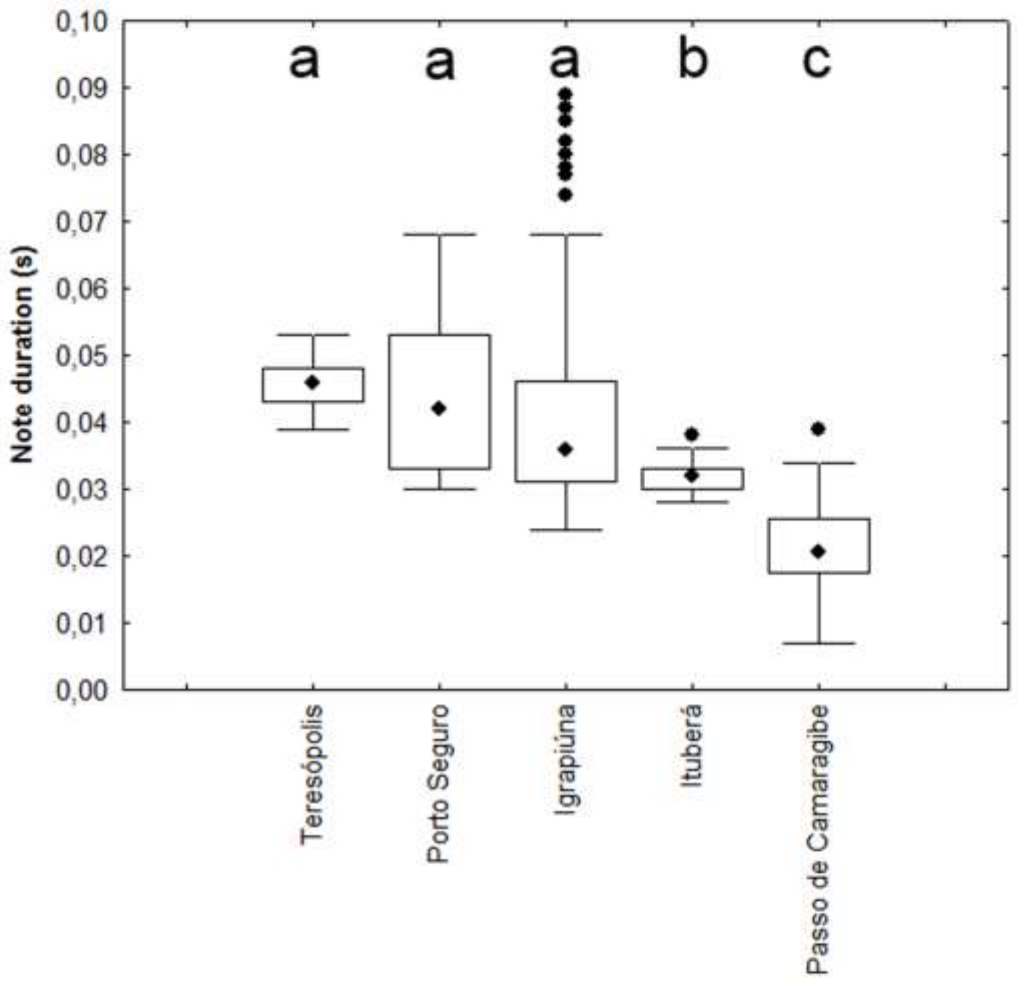

Figure S1. Note duration variation among Atlantic Forest Rocket Frogs populations. Box plot configuration = diamond: median, whiskers: non-outlier ranges, circles: outliers, box: 25% - 75% of data distribution. Letters a, b and c indicate statistical differences.
